# Patient-Based Assessment of Effectiveness of Voice Therapy in Vocal Mass Lesions with Secondary Muscle Tension Dysphonia

**Published:** 2018-05

**Authors:** Ahmad Reza Khatoonabadi, Hassan Khoramshahi, Seyyedeh Maryam Khoddami, Payman Dabirmoghaddam, Noureddin Nakhostin Ansari

**Affiliations:** 1 *Department of Speech Therapy, School of Rehabilitation, Tehran University of Medical Sciences, Tehran, Iran.*; 2 ***Musculoskeletal Rehabilitation*** ***Research Center, Ahvaz Jundishapur University of Medical Sciences, Ahvaz,*** ***Iran; **** Department of Speech Therapy, School of Rehabilitation, Tehran University of Medical Sciences, Tehran, Iran.*; 3 *Otorhinolaryngology Research Center, Tehran University of Medical Sciences, Tehran, Iran.*; 4 *Department of Physiotherapy, School of Rehabilitation, Tehran University of Medical Sciences, Tehran, Iran; *; 5 *Sports Medicine Research Center, Tehran University of Medical Sciences, Tehran, Iran.*

**Keywords:** Muscle tension dysphonia, Voice handicap index, Vocal mass lesions, Voice therapy

## Abstract

**Introduction:**

Use of patient-based voice assessment scales is an appropriate method that is frequently used to demonstrate effectiveness of voice therapy. This study was aimed at determining the effectiveness of voice therapy among patients with secondary muscle tension dysphonia (MTD) and vocal mass lesions.

**Materials and Methods:**

The study design was prospective, with within-participant repeated measures. Thirty-three patients with secondary MTD and vocal mass lesions participated in the study, selected on the basis of voice history, laryngeal palpation, and videostroboscopy examination. An experienced otolaryngologist and one experienced speech language pathologist undertook the diagnostic process. Voice therapy included both direct and indirect techniques and lasted approximately 2 months for all included patients. The voice handicap index (VHI) was used to evaluate the effectiveness of voice therapy among included patients. Paired* t*-test, size of the standardized effect (ES_I_), and mean standardized response (ES_II_) were used to analyze effectiveness of the target voice therapy.

**Results:**

The findings of this study indicate a statistically significant improvement after the voice therapy protocol (P<0.05; t>1.96). Results of ES_I_ and ES_II _demonstrate that the VHI scale is sufficiently responsive to detect voice therapy change (ES>0.8).

**Conclusion:**

This study recommends a combination of direct and indirect voice therapy in the vocal rehabilitation of patients with secondary MTD and vocal mass lesions. Furthormore, we recommend use of the VHI scale to show voice therapy changes for both clinical and research purposes.

## Introduction

Voice assessment scales evaluate the voice as a multi-dimensional phenomenon. Five aspects of voice quality are patient-based, auditory-perceptual, acoustic, aerodynamic, and the visual method (1–5). The patient-based assessment is an appropriate way to indicate quantity and quality of progress in voice therapy. The patient-based scale has additional information compared with other voice assessment scales (6,7).

The voice handicap index (VHI) is a patient-based voice scale that has frequently been used for both clinical and research purposes. High reliability and validity of the VHI have been demonstrated across several languages (6,7). In 2013, Moradi et al. confirmed the validity and reliability of the Persian version of this scale for Iranian subjects (6). Three subscales contribute to the VHI assessment. The physical component indicates the perception of patients regarding discomfort and the voice, the emotional subscale evaluates the emotions that patients may have about the disorder, and the functional aspect analyzes the influence of the disorder on performing activities of daily life (6–8).

Voice assessment scales are used to detect the effect of voice therapy approaches among different voice disorders. The VHI is a validated voice-related quality of life (QoL) questionnaire that is used individually to evaluate treatment effectiveness in benign voice disorders (e.g., vocal fold nodules, vocal fold polyps, and vocal fold polyps) (9–12). Results from studies conducted by Rosen et al. and Craig et al. show that VHI is applied for the evaluation of treatment efficacy in voice disorders (9,12), especially in patients with muscle tension dysphonia (MTD). Furthermore, some studies have used this questionnaire with other voice assessment scales, such as auditory-perceptual scales and videostroboscopy for this purpose (13,14).

Vocal mass lesions are a group of abnormal unilateral or bilateral lesions within or along the mid-membranous part of the vocal fold that is located within the superficial lamina propria (15,16). These vocal fold lesions generally appear in the form of nodules, polyps, and cysts (17,18). They present with MTD, and their symptoms are voice fatigue, dryness, narrowed vocal range, and disordered voice quality (15,16). However, MTD is one of the most prevalent functional voice disorders (19-21), and can be either primary or secondary. Primary MTD is described as a voice disorder in the absence of a clear laryngeal pathology with visible or palpable tension or stiffness in the neck, jaw, shoulder, and throat. The maladaptive compensatory voice behaviors adopted in response to laryngeal pathological conditions such as benign lesions, paresis, or other disturbances are related to secondary MTD. Therefore, vocal mass lesions are more likely to present with secondary MTD (19-21). According to the literature, some studies have evaluated the effect of voice therapy alone or in combination with laryngeal manual therapy in patients with MTD. Direct and indirect voice therapy is suggested for MTD patients in articles (21-33).

Clinicians need to be able to show their patients’ voice progress after voice therapy. It is also important to consider the patients’ beliefs about the effect of their voice treatment (2). It is also considerable to both the therapist and patient to be knowledgeable concerning which voice therapy approaches are effective to detect voice therapy improvement. This study aimed at determining the effectiveness of voice therapy among patients with different vocal mass lesions with secondary MTD. The second goal was to investigate the responsiveness of the VHI scale to voice therapy.

## Materials and Methods

**Fig 1:**
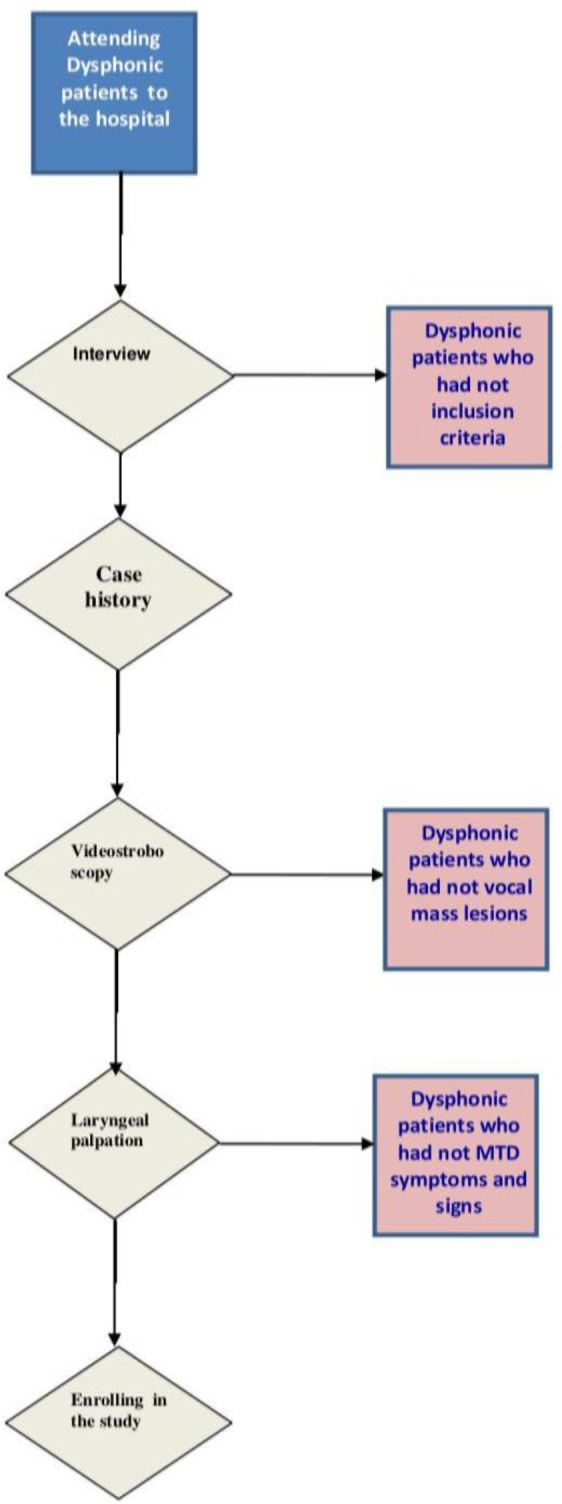
Decision ﬂowchart summarizing enrollment of patients in this study

Thirty-three patients (10 males, and 23 females) with vocal mass lesions due to secondary MTD who attended ENT clinics at Amir Alam Hospital in Tehran, Iran participated in this study. Vocal fold nodules, polyps, and cysts were considered as signs of MTD for inclusion in the study. Subjects were evaluated by an experienced otolaryngologist (with at least 5 years of experiences in voice field) and one experienced speech language pathologist (SLP) based on case history, laryngeal palpation, and visual examination. After an interview with each dysphonic patient, and based on their voice history, those with complaints of dysphonia were further evaluated for secondary MTD symptoms. The videostroboscopy examination was performed to assess the vocal mass lesions of the participants. Supra-glottic constrictions and posterior chink of false vocal folds were also evaluated in some videostroboscopic characteristics of MTD. The Angsuwarangsee and Morrison palpation examination (33) was performed to indicate laryngeal tension (Fig.1). 

This scale uses a palpation approach to evaluate tension in the supra-hyoid, thyrohyoid, muscles and the cricothyroid area.

The patients ranged from 21 to 55 years of age, and had vocal folds nodules, polyps, or cysts related to secondary MTD that were diagnosed by a videostroboscopic examination and laryngeal palpation. History of voice therapy, larynx surgery, asthma, taking drying medications, allergy, or any other concomitant health problems**,** hearing impairments, and inability to read were exclusion criteria of the present study. Patients who had inability to follow instructions during voice therapy because of low literacy and impatience were also excluded.

*Ethical considerations:*

The Ethics Committee at Tehran University of Medical Sciences (TUMS), Tehran, Iran approved the protocol, and approval was granted by the Institutional Review Board in the School of Rehabilitation, at TUMS, Tehran, Iran. Moreover, after explaining the aims of the study to each patient, written consent was obtained from each participant, and patients had the right to withdraw from the study at any time.

Current study was prospective with repeated measures. Included Participants were selected from patients who presented with a complaint of dysphonia at the ENT clinic at Amir Alam Hospital in Tehran. The videostroboscopic examination and questionnaires that included the Persian version of the VHI was completed by one SLP and another ENT. Finally, a patient-based assessment using the VHI was repeated after the treatment. Pre-therapy and post-therapy evaluations and voice therapy were performed by the SLP.

Based on evidence-based practice, voice therapy in patients with vocal mass lesions due to secondary MTD consisted of direct and indirect approaches(28–39) performed by an experienced SLP who was not blinded to the collection and analyses of the data. Indirect voice therapy consist of hydration, avoidance of smoking, avoidance of vocal fatigue, vocal abuse, and behavioral anti-reflux regimens. Direct voice therapy consist of chewing, yawn-sigh, correction of general body posture, training in abdominal breathing, Um-Hum, and inhalation phonation as well as circum-laryngeal manual therapy (CMT) (21–34). CMT therapy and symptom-based therapy performed as a physiological treatment. The voice therapy was given for 50 min in every session, twice a week for a total of 16 sessions over 2 months. Voice therapy exercises were practiced by patients at home. Correction of general body posture, training on abdominal breathing and chewing were presented in the first session of therapy. Based on the severity of MTD and the type of vocal mass lesions, a decision concerning the order of other direct voice therapies was made. SPSS statistics V 22.0 (SPSS Inc., Chicago, IL, USA) was used for statistical tests. The paired* t*-test was performed to show the effect of voice therapy after treatment among the studied patients. We used the size of the standard effect or effect size and mean standardized response (MSR) based on the mean and standard deviation of the effect and pre-therapy data. These two analyses were introduced as ES_I,_ and ES_II_, respectively. The size of the standardized effect was measured by dividing the mean differences between the pre-therapy and post-therapy scores to the standard deviation (SD) of the pre-therapy values. The MSR was computed by dividing the mean of the differences between the pre-therapy and the post-therapy values (change) to the SD of the change (34–41). The values 0.20, 0.50, and 0.80 or higher indicate small, moderate, and high sensitivity or responsiveness, respectively, to the therapy effect (35–37). Moreover, t-values more than 1.96 demonstrated a statistically significant effect of treatment and high responsiveness to change of the target scale (36-37). Like other responsiveness studies, we first assessed the effectiveness of the voice therapy by *t*-test, then based on the results of ES_I,_ and ES_II_ we evaluated the responsiveness of the VHI scale (35–40).

## Results

Thirty-six patients were evaluated in the pre-therapy stage, of whom 33 completed the treatment program. Patients with higher values on the VHI were more likely to leave the study. Because of the length of the voice therapy program (i.e., 2 months) and the slow rate of improvement, three of the included patients left the study. The mean age of the patients was 39±8 years (range, 21–54 years). The duration of the voice disorder ranged from 4 to 9 months (Table.1).

**Table 1:** Demographic characteristics of participants (n=33)

	**Age (Year)**				**Duration of disorder (Month)**			
	**Mean**	**SD**	**Min**	**Max**	**Mean**	**SD**	**Min**	**Max**
Nodules group (1 male, 10 female)	37	7	25	51	6	1	4	8
Polyp group (6 male, 5 female)	36	8	21	52	6	1	4	7
Cyst group (3 male, 8 female)	43	9	28	54	7	1	5	9
Total	39	8	21	54	6	1	4	9

The outcomes of the measures of interest are presented in Table 2. The mean of the functional subscale decreased from 17 to 7 over two therapy occasions. Similar to the functional subscale, the mean pre-therapy values changed from 22 to 11 for the physical subscale, and from 55 to 26 for the emotional subscale.

**Table 2:** Pre-therapy and post-therapy values of VHI

	**Pre-therapy values**				**Post-therapy values**			
	**Mean**	**SD**	**Min**	**Max**	**Mean**	**SD**	**Min**	**Max**
Functional subscale	17	9	1	37	8	6	0	25
Physical subscale	22	7	8	37	11	6	3	28
Emotional subscale	15	8	1	37	6	6	0	17
Total score	55	23	15	99	26	16	4	63

VHI: Voice Handicap Index

The paired* t*-test results shown in Table 3 demonstrate a statistically significant change in all three subscales and total score of VHI over time (P<0.05; t>1.96). Furthermore, ES_I_ and ES_II_ analyses indicated that the highest values of ES_I_ were observed for the physical subscale (ES_1_=1.57), while the lowest value for the functional and emotional subscales was 1. ES_II_ values were 1.8 for the physical subscale and 1.05 for the total score.

**Table 3:** Effectiveness of voice therapy indicated by VHI

	**(Paired ** **t** **-test)**	**ES**_I_	**ES**_II_
Functional subscale	P<0.05; t=7	1	1.2
Physical subscale	P<0.05; t=9	1.57	1.8
Emotional subscale	P<0.05; t=7	1	1.3
Total score	P<0.05; t=8	1.02	1.05

ES: Effect Size

## Discussion

The current study investigated the effectiveness of voice assessment scales among patients with vocal mass lesions due to secondary MTD. Voice assessments can detect an effect of therapy among patients. Studies of the effectiveness of commonly used voice assessments such as the VHI are critical because they clearly show the effect and sensitivity of changes due to voice therapy.

The main objective of our study was to evaluate the effect of voice therapy among patients with vocal mass lesions due to secondary MTD. 

To address this issue, we used the target patient-based assessment scale (the Persian version of VHI) in patients with vocal mass lesions due to secondary MTD, before and after voice therapy. Several studies have previously focused on the effect of voice therapy in functional voice disorders. According to these articles, direct and indirect voice therapy was used for the vocal rehabilitation of patients with MTD as a functional voice disorder. Based on evidence-based practice, we performed a combination of voice therapy with manual therapy in our patients.

The VHI scale helps to evaluate the effect of voice disorders on different aspects of the patients’ daily lives, such as physical, emotional, and functional aspects (8). Results of the present study indicate that the Persian version of the VHI was able to show the effectiveness of voice therapy in vocal mass lesions with secondary MTD. This result is consistent with the results of Rosen et al., Stuut et al., Craig et al., and Bouwers and Drikkers; all of whom used the VHI scale to evaluate the efficacy of voice therapy in MTD patients with benign voice disorders (e.g., nodules, polyps, and cysts). Before initiation of voice therapy, the mean total VHI score was greater than the cut-off point for the Persian version (total score >14.5) (6). This indicates that the voice disorders affected the daily lifestyle of all patients involved in our study. Moreover, the mean value of the physical subscale was greater than the two other subscales, indicating that the studied patients were more affected physically than functionally or emotionally.

Based on the paired *t*-test results, all patients who participated in our study demonstrated an effect of voice therapy by the Persian version of the VHI questionnaire (P>0.05). Consistent with the findings of previous studies that reported on the effectiveness of a combination of direct and indirect voice therapy in functional voice disorders, our findings indicate that our voice therapy protocol was effective in vocal mass lesions in patients with secondary MTD.

Results of the paired t-test demonstrate that a statistically significant effect of voice therapy occurred over time, while a high responsiveness to change was shown for the Persian version of the VHI (t>1.96). Furthermore, ES_I_ and ES_II_ analyses demonstrated high responsiveness for VHI (ES>0.8). 

These findings are consistent with studies that indicate high reliability, validity, and responsiveness to change of VHI (6,7,41). Therefore, the results of the current study recommend the Persian version of the VHI as a sensitive scale with which to indicate vocal rehabilitation progress in vocal mass lesions with secondary MTD.

## Conclusion

The present study revealed that the patient-based scale of interest detected an effect of voice therapy among patients with vocal mass lesions due to secondary MTD. Values of the Persian version of the VHI suggest that the subscales and total score of VHI are sensitive to voice therapy changes. None of the target voice assessments were perfect, and they could not predict changes in all five aspects of voice quality; each evaluates only one aspect of voice quality. Thus, performing a complete assessment of voice quality is the best model for evaluating improvement in voice quality. Furthermore, values of the subscales of VHI were different, while the highest deviance was seen for the physical aspect. Our study focused only on one voice assessment scale and the special voice therapy protocol. Further studies on other voice assessment scales in a large sample may provide a better demonstration of the effectiveness of voice therapy.
